# Cost analysis of a sequential antimicrobial therapy protocol in patients with pneumonia: A multicenter study

**DOI:** 10.1371/journal.pone.0354476

**Published:** 2026-07-28

**Authors:** Flaviane Gecler Parreira, Juan Vicente da Cunha Leal, Aline Correa de Araujo, Amanda Mota Conceição, Amanda Migliorini do Nascimento, Carolina Serra Cabo Jogaib, Sabrina Calil-Elias, Jéssica Pronestino de Lima Moreira

**Affiliations:** 1 PPG GAFAR – Faculdade de Farmácia da Universidade Federal Fluminense, Niterói, Rio de Janeiro, Brazil; 2 Faculdade de Farmácia da Universidade Federal Fluminense, Niterói, Rio de Janeiro, Brazil; 3 Hospital Monte Klinikum, Fortaleza, Ceará, Brazil; 4 Hospital Samaritano Higienópolis, São Paulo, São Paulo, Brazil; 5 PPG CAPS - Faculdade de Farmácia da Universidade Federal Fluminense, Niterói, Rio de Janeiro, Brazil; Roche Pharma China, CHINA

## Abstract

Pneumonia is one of the leading causes of hospitalization and mortality due to infectious diseases and is also responsible for a substantial proportion of antimicrobial prescriptions. This study aimed to analyze the clinical outcomes and the economic implications associated with a sequential antimicrobial in hospitalized patients with pneumonia. This was a multicenter, retrospective, observational cost analysis study conducted in four private Brazilian hospitals between January 2022 and December 2023. A total of 185 medical records from patients undergoing the sequential therapy protocol were included. Clinical outcomes, length of hospital stay, acceptability among physicians of pharmacist-led interventions for switching the route of administration, and the direct costs related to medications and human resources were assessed according to a microcosting methodology. Among the included patients, 74.6% transitioned to the oral route, and 88.4% showed a positive clinical outcome. Sequential therapy was associated with a significant reduction in mean length of stay (10.1 vs. 14.7 days; p = 0.024) and was associated with 55% lower treatment costs, corresponding to an estimated cost avoidance of US$ 4,529.96. The findings suggest that sequential antimicrobial therapy is an safe and economically advantageous strategy that contributes to the rational use of antimicrobials and the optimization of hospital resources.

## Introduction

Pneumonia is an acute respiratory infection that affects the pulmonary parenchyma and is frequently observed in children under five years of age and in adults older than 75 years. It may occur in different settings, including community-acquired and healthcare-associated pneumonia [1,2].

This condition is the leading cause of hospitalization and mortality among infectious diseases and the most frequent reason for antimicrobial prescriptions in adults [3–5]. Data from the Global Point Prevalence Survey (Global-PPS) indicate that lower respiratory tract infections, including pneumonia, accounted for 19,2% of indications for antimicrobial use worldwide and 29,2% in Brazil [6].

Although most hospitalized patients with pneumonia initially receive intravenous antimicrobial therapy [7], the oral route should be preferred whenever possible [8]. International guidelines, including those from the Infectious Diseases Society of America/American Thoracic Society (IDSA/ATS) and the National Institute for Health and Care Excellence (NICE), recommend initiating intravenous antimicrobial therapy in hospitalized patients with moderate to severe pneumonia. Transition to oral therapy may be considered when the patient shows clinical improvement, is hemodynamically stable, and is able to tolerate oral medications [9,10].

For IV-to-oral antimicrobial switching to be safe and effective, predefined criteria must be followed to determine patient eligibility. These criteria include hemodynamic stability, absence of fever, absence of leukopenia, normal oral intake, and a functioning gastrointestinal tract [11–13]. In addition, the antimicrobial agent must be available as an oral formulation and have adequate oral bioavailability [14,15].

Sequential antimicrobial therapy offers several advantages, including shorter hospital stays, fewer adverse events, primarily because intravenous catheter use is avoided, improved patient comfort, and reduced nursing workload [16–19].

Switching the route of administration also results in relevant economic impacts. Studies have shown that sequential antimicrobial therapy not only maintains treatment effectiveness but also optimizes resource utilization, resulting in clinical and economic benefits [20–23].

In Brazil, the care of patients with pneumonia generally begins in emergency departments, which represent the main entry point to the healthcare system. At this stage, clinical evaluation, diagnostic confirmation, and severity stratification are performed. Patients with mild disease may be managed on an outpatient basis, whereas those with moderate to severe presentations are generally hospitalized for intravenous antimicrobial treatment and clinical monitoring [24]

Although there is evidence that the use of oral antimicrobials for the treatment of pneumonia is as effective as intravenous therapy [25–27], systematic studies in Brazil remain scarce regarding the effectiveness of sequential antimicrobial therapy, particularly in terms of clinical outcomes, impact on length of stay, and measurement of avoided costs.

This study aimed to analyze the clinical outcomes and economic impact of the sequential antimicrobial therapy protocol used for the treatment of pneumonia in Brazilian hospitals.

## Methods

This was a multicenter, retrospective, observational study with a cost-avoidance analysis, conducted in four hospitals in the private healthcare sector located in different Brazilian states between 01/01/2022 and 31/12/2023. The sequential antimicrobial therapy protocol used in the present study was developed based on the protocol described by McLaughlin [11].

The protocol established that pharmacists should monitor patients receiving antimicrobials to identify those eligible for sequential therapy. Forty-eight hours after the initiation of intravenous therapy, the protocol was applied based on previously defined eligibility criteria: hemodynamic stability, absence of gastrointestinal tract impairment, and absence of neutropenia. Once these criteria were met, the pharmacist recommended that the medical team transition the patient from intravenous to oral therapy.

Data were collected from electronic medical records based on medical prescriptions, pharmacist documentation, and the clinical and epidemiological information management systems of the participating institutions.

Medical records of patients aged ≥18 years who underwent the sequential antimicrobial therapy protocol during the study period were included. Inclusion was restricted to adults because of significant differences between pediatric and adult patients regarding infection epidemiology, clinical management, and antimicrobial pharmacokinetics. Medical records with incomplete data for the variables of interest were excluded.

During the study period, 421 patients underwent the sequential antimicrobial therapy protocol. After screening, 185 patients with a diagnosis of pneumonia were included in the analysis. No medical records were excluded due to missing data or inconsistencies, as all records showed adequate completeness for the analysis of the variables of interest.

Clinical and demographic variables included hospital ward, age, sex, antimicrobial susceptibility testing, antimicrobial agent, dose, expected treatment duration, duration of intravenous treatment, duration of oral treatment, acceptance of pharmacist interventions for switching the route of administration, length of hospital stay, and clinical outcome. Research Electronic Data Capture (REDCap®) software, version 14.5.8, was used for data entry and management. Data collection was performed between 01/09/2024 and 31/12/2024.

The comparison between patients who transitioned from intravenous to oral therapy and those who remained on exclusively intravenous treatment was defined based on acceptance of the pharmacist intervention by the medical team. There was no prior separation between the exclusive IV and IV-to-oral groups at the time of patient inclusion, since all eligible patients underwent the same evaluation protocol for sequential therapy. Patients who remained on exclusively intravenous treatment were clinically eligible for route conversion, but the medical team did not accept the pharmacist’s recommendation to switch to oral therapy.

The acceptance rate of pharmacist recommendations for antimicrobial route conversion was then evaluated. An intervention was considered accepted when the medical prescription was modified, with effective replacement of intravenous therapy by oral therapy.

Negative outcomes were defined as therapeutic failure after transition to oral antimicrobial therapy, including clinical worsening, increased C-reactive protein levels, or a new infectious focus requiring resumption of intravenous therapy, as well as death. Positive outcomes were defined as clinical improvement, evidenced by resolution or regression of the signs and symptoms of infection, followed by hospital discharge.

Hospital length of stay was also analyzed by comparing patients who transitioned to oral therapy with those who received exclusively intravenous treatment throughout the treatment period. Length of stay was defined as the period between hospital admission and discharge. Medical records of patients with hospital stays longer than 60 days were excluded from this analysis because they were considered outliers and frequently reflected the presence of associated comorbidities or clinical conditions not directly related to pneumonia. Inclusion of these cases could overestimate the mean hospital stay, compromise comparability between groups, and hinder a more accurate assessment of the impact of sequential therapy on length of stay.

The economic evaluation consisted of a cost-avoidance analysis, which was performed using a microcosting approach in accordance with the guidelines issued by the Secretariat of Science, Technology, Innovation, and Strategic Health Inputs for microcosting studies applied to health economic evaluations [28]. A bottom-up approach was adopted, including only direct medical costs related to medications and personnel. Cost estimation was performed through the identification, measurement, and valuation of each resource used, including medications, materials, and personnel. Two scenarios were compared: full intravenous antimicrobial therapy, corresponding to the expected cost considering the projected treatment duration, and therapy involving replacement of intravenous antimicrobials with oral therapy, corresponding to the actual cost.

Initially, the antimicrobial administration process was mapped, with detailed description of all activities performed. This mapping was developed based on a review of institutional protocols for medication preparation and administration. Based on this mapping, cost-generating activities related to medication preparation and administration performed by healthcare professionals were identified for the microcosting analysis. The identified activities were: (1) preparation of intravenous medication, including reconstitution and dilution, for antimicrobials requiring reconstitution followed by dilution; (2) preparation of intravenous medication, including dilution, for antimicrobials requiring dilution only; (3) preparation of ready-to-use intravenous medication, supplied as an infusion bag; (4) administration of intravenous antimicrobials; (5) preparation of oral medication; and (6) administration of oral medication.

Subsequently, the individuals involved in the process were identified and classified as personnel resources. The estimated time required for activities performed by healthcare professionals was obtained through audits of medication preparation and administration. For analysis purposes, the mean execution time of each stage of the process was used.

In the subsequent phase, personnel costs were estimated. Unit personnel costs were calculated based on salaries plus payroll charges, using data provided by the institutions and December 2023 salaries as the reference. Considering the monthly workload, hourly and per-minute remuneration of professionals were calculated. Subsequently, the total personnel cost was obtained by multiplying the per-minute cost by the mean time spent on each activity and by the frequency with which the activity was performed per patient.

The resources consumed by patients were identified through review of medical prescriptions and electronic medical records. Subsequently, the quantity of each resource used was calculated, including medications, diluents, syringes, and needles. For valuation purposes, acquisition prices corresponding to the month of consumption, obtained from the hospital management system, were considered.

Finally, the total treatment cost was estimated by summing expenditures on personnel and medications. Patients who resumed intravenous antimicrobial therapy were excluded from this analysis. The avoided cost associated with sequential therapy was recorded in Brazilian Reais (BRL) and subsequently converted to U.S. dollars (US$) according to the official exchange rate published by the Central Bank of Brazil on May 9, 2025, when US$ 1.00 = BRL 5.65 [29].

A descriptive analysis was performed to characterize the study population. Variables were expressed as absolute and relative frequencies. Histogram analysis and the Kolmogorov-Smirnov test were used to assess normality. The Mann-Whitney test was used for numerical variables, whereas Pearson’s chi-square test or Fisher’s exact test was used to evaluate associations between categorical variables. Bivariate analyses were performed to assess which variables were associated with outcomes and with the acceptability of pharmacist interventions for route conversion.

Multiple binary logistic regression was used to identify factors associated with outcomes. Reference categories (OR=1) were defined based on the theoretically established baseline category. A statistical significance level of 5% was adopted, and associations whose 95% confidence interval did not include the value 1 were considered statistically significant.

To compare the expected costs of exclusively intravenous treatment with the costs actually observed after transition to oral administration, the nonparametric paired Wilcoxon test was applied. A significance level of 5% was considered for all analyses. Statistical analysis was performed using Statistical Package for the Social Sciences (SPSS®) software, version 29.

This study was approved by the Research Ethics Committee of the Universidade Federal Fluminense (approval number 6.738.462) and by the ethics committees of the participating hospitals. The requirement for informed consent was waived due to the retrospective nature of the study and the use of anonymized data.

## Results

A total of 185 medical records that met the study eligibility criteria were reviewed. Of these, 138 (74.6%) patients transitioned from intravenous to oral therapy, corresponding to the IV-to-oral group, whereas 47 (25.4%) remained on exclusively intravenous treatment, corresponding to the IV group, due to the medical team’s non-acceptance of the pharmacist intervention recommending route conversion.

Considering that patient allocation into groups was determined by the medical team’s acceptance of the intervention, patient characteristics stratified by treatment group are presented in Table 1.

**Table 1:** Sociodemographic and clinical profile of patients with pneumonia included in the sequential therapy protocol, 2022–2023. Brazil, 2025.

Variables	Total (n = 185)		Acceptability				p-value^a^
			Accepted IV-VO (n = 138)		Not accepted IV only (n = 47)		
	n	%	n	%	n	%	
**Sex**							0,047
Male	82	44,3	67	81,7	15	18,3	
Female	103	55,7	71	68,9	32	31,1	
**Age**							0,902
18 a 59 years	23	12,4	17	73,9	6	26,1	
60 a 79 years	76	41,1	58	76,3	18	23,7	
> 80 years	86	46,5	63	73,3	23	26,7	
**Hospital Unit**							0,330
Intensive Care Unit (ICU)	91	49,2	65	71,4	26	28,6	
Inpatient Unit	94	50,8	73	77,7	21	22,3	
**Antimicrobial Agent**							
Azithromycin	107	57,8	86	80,4	21	19,6	
Ciprofloxacin	1	0,5	0	0	1	100	
Cefuroxime	3	1,6	2	66,7	1	33,3	
Clarithromycin	25	13,5	21	84,0	4	16,0	
Clindamycin	14	7,6	8	57,1	6	42,9	
Fluconazole	5	2,7	1	20,0	4	80,0	
Levofloxacin	23	12,4	14	60,9	9	39,1	
Linezolid	3	1,6	3	100,0	0	0,0	
Moxifloxacin	2	1,1	1	50,0	1	50,0	
Sulfamethoxazole + Trimethoprim	1	0,5	1	100,0	0	0,0	
Voriconazole	1	0,5	1	100,0	0	0,0	
**Death**							0,681
Yes	7	3,8	6	85,7	1	14,3	
No	178	96,2	132	74,2	46	25,8	

^a^Pearson’s Chi-square Test or Fisher’s Exact Test.

Regarding sex, a statistically significant difference was observed (p = 0.047), with higher acceptability among male patients (81.7%), whereas acceptability among female patients was 68.9%. Acceptability across age groups was similar, with no statistically significant association observed (p = 0.902).

Regarding hospitalization setting, 94 (50.8%) patients were admitted to inpatient wards, of whom 73 (77.7%) transitioned to oral therapy. Among the 91 patients admitted to the Intensive Care Unit (ICU), 65 (71.4%) transitioned to oral antimicrobial therapy. No statistically significant association was observed between hospitalization setting and acceptability (p = 0.330).

The IV and IV-to-oral groups showed similar distributions for most demographic and clinical variables evaluated, although a statistically significant difference was observed for sex.

Among the 185 patients included in the study, 7 (3.8%) died. Of these, only one patient did not transition to oral therapy. Although the proportion of deaths was higher among patients who received oral treatment, this difference was not statistically significant (p = 0.681), precluding robust inferences regarding an association between oral therapy and mortality.

Table 2 presents the results related to the outcome following the switch in the antimicrobial route of administration. A positive outcome was defined as clinical improvement, evidenced by the resolution or regression of signs and symptoms of infection, followed by hospital discharge. A negative outcome was defined as therapeutic failure, characterized by the need to resume intravenous antimicrobial therapy or by death. A positive outcome was observed in 88.4% of patients.

**Table 2:** Sociodemographic and clinical profile of patients included in the sequential antimicrobial therapy protocol in 2022–2023. Brazil, 2025.

Variables	Total (n=)		Outcome of Sequential Therapy				p-value^a^
			Positive (n = 122)		Negative (n = 16)		
	n	%	n	%	n	%	
**Sex**							0.512
Male	67	48.6	58	86.6	9	13.4	
Female	71	51.4	64	90.1	7	9.9	
**Age**							0.756
18–59 years	17	12.3	15	88.2	2	11.8	
60–79 years	58	42.0	50	86.2	8	13.8	
> 80 years	63	45.7	57	90.5	6	9.5	
**Hospital Unit**							0.189
Intensive Care Unit (ICU)	65	47.1	55	84.6	10	15.4	
Inpatient Unit	73	52.9	67	91.8	6	8.2	
**Antimicrobial Agent**							
Azithromycin	86	62.3	83	96.5	3	3.5	
Cefuroxime	2	1.4	1	50.0	1	50.0	
Clarithromycin	21	15.2	19	90.5	2	9.5	
Clindamycin	8	5.8	7	87.5	1	12.5	
Fluconazole	1	0.7	1	100.0	0	0.0	
Levofloxacin	14	10.1	10	71.4	4	28.6	
Linezolid	3	2.2	0	0.0	3	100.0	
Moxifloxacin	1	0.7	0	0.0	1	100.0	
Sulfamethoxazole + Trimethoprim	1	0.7	1	100	0	0.0	
Voriconazole	1	0.7	0	0.0	1	100.0	

^a^Pearson’s Chi-square Test or Fisher’s Exact Test.

Bivariate analysis was performed to evaluate the association between oral therapy and outcomes. Positive outcomes were observed in 86.6% of men and 90.1% of women. No statistically significant association was observed between sex and outcomes (p = 0.512). Age group did not influence the success rate of sequential therapy, with no statistically significant difference identified (p = 0.756). Among patients admitted to the ICU (84.6%) and inpatient wards (91.8%), no statistically significant association was identified between hospitalization setting and positive outcome (p = 0.189).

Table 3 presents the adjusted odds ratio (OR) for negative outcomes (n = 16) associated with sequential antimicrobial therapy according to sociodemographic and clinical variables. Male patients had an adjusted OR of 0.76 (95% CI: 0.262–2.201) compared with female patients. Although not statistically significant, the point estimate suggested an approximately 24% lower likelihood of negative outcomes among men.

**Table 3:** Adjusted odds ratio for negative outcomes (n = 16), according to sociodemographic and clinical variables, 2022–2023. Brazil, 2025.

Variables	ORadjusted	IC95%_adjusted_
**Sex**		
Male	0,76	0,262 − 2,201
Female	1	
**Age**		
18 a 59 years	1.153	0,208 − 6,382
60 a 79 years	1.507	0,483 − 4,695
> 80 years	1	
**Hospital Unit**		
Intensive Care Unit (ICU)	0,497	0,169 − 1,467
Inpatient Unit	1	

Regarding the reference age group, older than 80 years, patients aged 18–59 years and 60–79 years had adjusted ORs of 1.153 (0.208–6.382) and 1.507 (0.483–4.695), respectively, suggesting a possible, but non-significant, increase in the likelihood of negative outcomes among younger age groups.

The point estimate suggested an approximately 50% lower likelihood of negative outcomes among ICU patients compared with ward patients, although this association was not statistically significant. Overall, none of the analyzed variables remained significantly associated with negative outcomes after model adjustment.

Patients who transitioned to oral therapy were associated with a shorter length of hospital stay (mean 10.1 days, SD = 9.2; median = 7) than those who remained on intravenous therapy (mean 14.7 days, SD = 14.0; median = 9). This difference was statistically significant (Mann-Whitney test, p = 0.024).

For the microcosting analysis, all resources used, including medications and personnel resources, were identified, measured, and valued. Two scenarios were compared: exclusive intravenous antimicrobial therapy and therapy involving transition from intravenous to oral administration.

The meantime spent on each activity was recorded. The activity that required the greatest amount of staff time was the preparation of intravenous antimicrobials requiring reconstitution and dilution, with a mean duration of 11 minutes per dose.

The cost related to medications and supplies was calculated individually. The acquisition cost of medications and materials used for administration was obtained from the hospital management system, according to the prices in effect during the study period (2022–2023). Because these prices may vary monthly, the purchase price recorded in the month in which each patient received the medication was used. The unit costs of medications and supplies were recorded and multiplied by the quantity consumed by each patient during treatment. The total cost was then calculated.

It was observed that the healthcare professional responsible for medication preparation and administration was the nursing technician. The cost of human resources was calculated based on the base salary plus benefits for the professional category and the monthly work schedule during the study period. The professional remuneration ranged from US$ 0.078 to US$ 0.030 per minute. The estimated cost of human resources was then calculated for each activity performed. The activity that required the greatest human resource expenditure was the preparation of intravenous antimicrobials requiring reconstitution and dilution, with a mean cost per dose ranging from US$ 0.331 to US$ 0.856.

Finally, the total treatment cost (actual cost) following the switch from intravenous to oral antimicrobial administration was calculated, as well as the expected cost if the entire treatment had been administered intravenously. The avoided cost was defined as the difference between the expected cost and the actual cost. An overall avoided cost of BRL 25,592.66 (US$ 4,529.96) was observed, representing an estimated cost avoidance equivalent to approximately 55% of the projected intravenous treatment cost.

**Fig 1:**
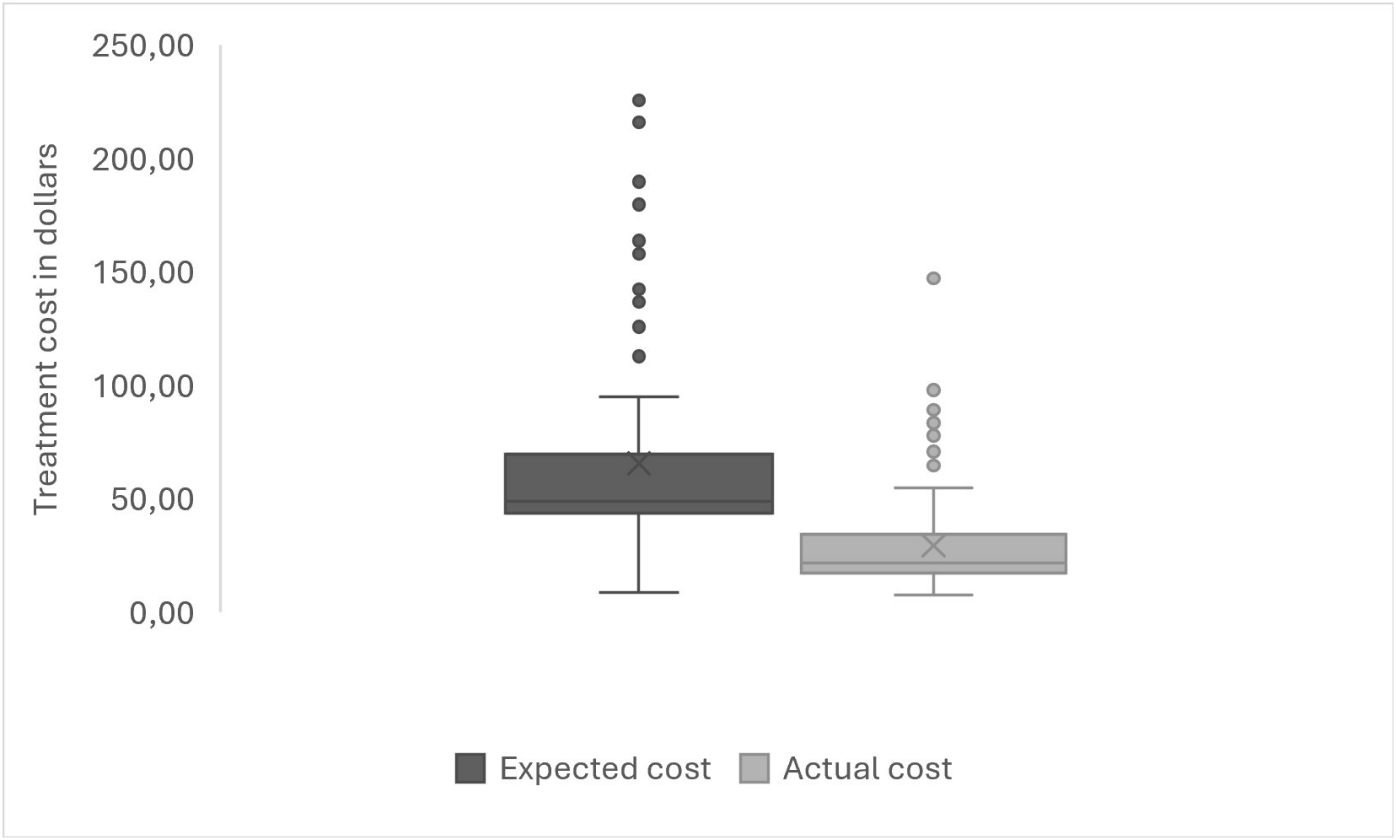
Analysis of antimicrobial treatment costs in dollars: expected versus actual.

Fig 1 presents the boxplot comparing the projected cost for exclusively intravenous treatment (expected cost) and the actual cost after switching from intravenous to oral administration. This comparison aimed to evaluate the impact of the sequential therapy protocol on cost reduction. A statistically significant difference (p < 0,001) was observed between the actual and expected costs, with the actual cost being significantly lower.

## Discussion

The findings suggest that the sequential antimicrobial therapy protocol was associated with favorable clinical outcomes, characterized by a predominance of favorable clinical progression and a low frequency of negative outcomes. In addition, implementation of the protocol was associated with lower treatment-related costs, supporting the feasibility of sequential therapy from both clinical and economic perspectives. These findings are consistent with the results previously reported by other studies, which also emphasized the safety, effectiveness, and cost reduction associated with oral therapy [30–32].

The acceptability rate of the pharmacist-led intervention for switching the antimicrobial route of administration was high. A similar result was observed by Nguyen et al. (2023) in a hospital in Vietnam [33] and by Mouwen et al. (2020) in a Dutch hospital [12].

However, prescribers’ lack of familiarity with the efficacy of oral antimicrobials in certain infections, as well as concerns that the oral route may not achieve adequate serum levels in patients with clinically severe conditions, are among the main factors compromising the consolidation of sequential therapy as an institutional practice and may interfere with acceptability [34,35]. Therefore, efforts should be directed toward education and guidance regarding the advantages of sequential antimicrobial therapy. Qualitative studies, such as that conducted by Zhang et al. (2024) [36], suggest that resistance to switching the route of administration may be related to the perception that oral therapy is less effective, although current evidence does not support this perception. When this perception is not addressed through updated clinical guidelines and continuing education, it may compromise the effectiveness of sequential therapy protocols.

Analysis of the clinical outcomes in the present study demonstrated that most patients undergoing oral therapy experienced positive outcomes. These results reinforce the safety of IV-to-oral transition, especially when strict clinical criteria are met, such as hemodynamic stability, absence of fever, laboratory improvement, and functional ability for oral intake. These findings are consistent with those reported by Buckel et al. (2017) [37] and Dinh et al. (2024) [38], which also support the feasibility and safety of sequential antimicrobial therapy in patients with pneumonia.

Among patients who experienced negative outcomes, 81% resumed intravenous therapy due to clinical or laboratory worsening, highlighting the importance of monitoring after route conversion, with continuous assessment of therapeutic response. Although therapeutic failure does not invalidate the strategy, it emphasizes that IV-to-oral transition should be accompanied by clinical surveillance. This reinforces the statement by Platts et al. (2022) [39] that clinical failure should be analyzed in light of infection progression.

Positive outcomes were observed regardless of hospitalization setting, whether ICU or inpatient ward, indicating that the safety of route conversion is not restricted to lower-complexity settings. This finding is consistent with the study conducted by Gasparetto et al. (2019) [20], which demonstrated that sequential therapy is also a safe strategy for critically ill patients admitted to the ICU.

The success of sequential therapy among older adults, particularly those older than 80 years, also deserves attention. The success rate of sequential therapy was similar to that observed in younger patients. Thus, the positive results among older adults reinforce the feasibility of the intervention when applied according to clear clinical criteria and with support from the multidisciplinary team. Alves et al. (2024) [40] further emphasized that sequential antimicrobial therapy in older patients is a safe alternative. Prolonged hospitalization among older adults increases the risk of functional decline, worsening of comorbidities, hospital-acquired infections, and reduced quality of life [41].

The absence of statistically significant associations between outcomes and sociodemographic and clinical variables suggests that the unfavorable events observed were probably attributable to patients’ baseline severity or comorbidities rather than to route conversion itself.

Multivariate analysis demonstrated that none of the sociodemographic or clinical variables evaluated showed a statistically significant association with negative outcomes related to sequential antimicrobial therapy after model adjustment.

The results of this study suggest that, provided well-defined criteria have been established, involving hemodynamic stability, absence of fever, and ability to ingest food, route switching can be safely adopted. This approach is consistent with Moehring et al. (2023) [42], who emphasizes the importance of clear guidelines to adequately identify patients eligible for route of administration transition, ensuring the efficacy and safety of the process.

In addition to favorable clinical outcomes, a relevant impact on healthcare indicators was also observed, particularly regarding hospital length of stay, which is considered one of the most relevant outcomes in the evaluation of sequential antimicrobial therapy effectiveness. The findings of the present study are consistent with those reported by Kaal et al. (2024) [27], Deshpande et al. (2023) [26], and Christensen et al. (2019) [43].

The analysis conducted by Broermann et al. (2023) [44] complements this discussion by identifying, through a linear regression model, the risks associated with prolonged hospitalization and showing that switching to oral antimicrobials was correlated with shorter hospital stays. Such reductions directly impact hospital efficiency by enabling better resource allocation and reducing patient exposure to hospitalization-related adverse events, such as nosocomial infections and complications associated with venous access (Gasparetto et al., 2019) [20]. The intervention discussed in the present study aligns with this principle by promoting rational resource utilization without compromising quality of care.

Sequential therapy was associated with lower treatment costs. The microcosting analysis conducted in this study demonstrated that replacing intravenous antimicrobial therapy with oral therapy associated with lower costs by slightly more than half compared with the estimated cost of full intravenous therapy. This finding reinforces the value of sequential therapy not only as a safety measure and a strategy for rational antimicrobial use, but also as an approach to healthcare cost optimization.

Notably, to date, no studies evaluating avoided costs associated with sequential antimicrobial therapy using a microcosting methodology have been identified in the literature. Therefore, the findings of the present study provide an original contribution to understanding the direct economic impact of this intervention, supporting decision-making and the improvement of institutional policies aimed at the rational use of antimicrobials.

## Limitations

Despite the promising results, this study has some limitations that should be considered when interpreting the data. First, this was a retrospective observational study, which limits rigorous control over variables and may introduce bias related to the quality of information recorded in electronic medical records. Although no statistically significant baseline differences were identified between the groups, the possibility of residual confounding inherent to retrospective observational studies cannot be completely excluded. It was also not possible to evaluate variables such as adherence to oral treatment after hospital discharge and post-discharge adverse events, which limits a comprehensive assessment of long-term clinical outcomes.

In addition, the economic evaluation consisted of a cost-avoidance analysis rather than a formal cost-effectiveness analysis. As this was a retrospective observational study, the observed economic differences should be interpreted as associations rather than causal effects attributable to sequential therapy.

## Conclusion

The results of this study support the feasibility, safety, and economic value of sequential antimicrobial therapy in hospitalized patients with pneumonia. The high rate of favorable clinical outcomes, combined with the significant reduction in length of stay and treatment-related costs, demonstrates the feasibility of switching from the intravenous to the oral route. These findings, particularly those related to microcosting analysis, provide novel evidence to support institutional decision-making and strengthen policies aimed at promoting the rational use of antimicrobials in hospital settings.

## Supporting information

S1 Table Costs of Antimicrobial and materials. (XLSX)

S2 Table Cost of the processes involved in preparing the medication. (XLSX)

S3 Table Calculation of expected cost (IV). (XLSX)

S4 Table Calculation of the actual treatment cost (IV-VO). (XLSX)
